# Prevalence and Outcome of Sepsis: Mortality and Prolonged Intensive Care Unit Stay among Sepsis Patients Admitted to a Tertiary Centre in Malaysia

**DOI:** 10.21315/mjms2023.30.6.12

**Published:** 2023-12-19

**Authors:** Kamaliah Azzma Kari, Wan Fadzlina Wan Muhd Shukeri, Najib Majdi Yaacob, Andrew Yunkai Li, Rhendra Hardy Zaini, Mohd Zulfakar Mazlan

**Affiliations:** 1 Department of Anaesthesiology and Intensive Care, School of Medical Sciences, Universiti Sains Malaysia, Kelantan, Malaysia; 2 Department of Anaesthesiology and Intensive Care, Hospital Universiti Sains Malaysia, Kelantan, Malaysia; 3 Biostatistics and Research Methodology Unit, School of Medical Sciences, Universiti Sains Malaysia, Kelantan, Malaysia; 4 Division of Respiratory and Critical Care Medicine, Department of Medicine, National University Hospital, National University Health System, Singapore

**Keywords:** sepsis, mortality, prolonged, ICU, stay

## Abstract

**Background:**

Sepsis and septic shock are the leading causes of critical care-related mortality worldwide. This study aimed to determine the prevalence of sepsis, its intensive care unit (ICU) mortality rate and the factors associated with both ICU mortality and prolonged stay.

**Methods:**

A prospective cohort study was conducted from January 2019 to December 2019 with adult patients presenting evidence of sepsis who were admitted to the ICU. Parameters were assessed in the ICU to determine the association with all-cause ICU mortality and prolonged stay.

**Results:**

Out of 607 adults, 292 with sepsis were admitted to the ICU in 2019, with a mean age of 50.98 (standard deviation [SD] = 17.75) years old. There was, thus, a 48% incidence of sepsis. Mortality was observed in 78 patients (mortality rate = 26.7%) (95% confidence interval [CI]: 21.7, 32.2). Patients with higher Glasgow coma scale (GCS) scores had lower odds of ICU mortality (adjusted odds ratio [OR] = 0.90; 95% CI: 0.82, 0.98; *P* = 0.019), while patients with higher sequential organ failure assessment (SOFA) scores had higher odds (adjusted OR = 1.22; 95% CI: 1.11, 1.35; *P* < 0.001). Eighty patients (37.4%) who survived had prolonged ICU stays (95% CI: 30.9, 44.2). Patients with higher albumin levels had lower odds of a prolonged ICU stay (adjusted OR = 0.94; 95% CI: 0.90, 0.98; *P* = 0.006) and patients on renal replacement therapy had higher odds of a prolonged ICU stay (adjusted OR = 1.25; 95% CI: 1.74, 7.12; *P* < 0.001).

**Conclusion:**

Our study identified a sepsis prevalence of 48% and an ICU mortality rate of 26.7% among adult patients admitted to the ICU. GCS and SOFA scores were the most important factors associated with ICU mortality.

## Introduction

Sepsis has traditionally been defined as life-threatening organ dysfunction resulting from a dysregulated host response to infection (1). Septic shock is sepsis without evidence of hypovolemia that requires vasopressor support to maintain a mean arterial pressure of 65 mmHg or more and serum lactate of 2 mmol/L or more (1–2).

The prevalence of sepsis varies from 13.6% to 39.3% in different regions (3). The incidence and mortality also differ globally, with the highest rates in sub-Saharan Africa, Oceania, South Asia, East Asia and Southeast Asia. The mortality rates in 2017 were 25.8% in Oceania and 35.3% in Africa, respectively (4). The intensive care unit (ICU) sepsis incidence in 2014 was 36.3 per 1,000 patient days and mortality was observed in 439 (55.7%) of 788 patients in Brazil (5).

A 90-day mortality rate of 35.5% was recorded in China (6), while it was 62.8% in India (7). In Turkey, it was reported as 55.7% for sepsis and 70.4% for septic shock (8). Apart from this, estimations of the mortality rate secondary to sepsis and septic shock in developing and low-income countries are scarce (9–11). An all-cause ICU mortality rate of about 18.3% was recorded in Malaysia (12). Meanwhile, in high-income countries, it is estimated to be between 17% and 26% (13–15).

An analysis of the Global Burden of Disease Study, which gathered data on 109 million patients from 1990–2017, identifies the following factors as associated with mortality: advanced age, higher simplified acute physiology score, previous immunosuppression, liver insufficiency, solid organ malignancy, renal failure and renal replacement therapy, higher acute physiological and chronic health evaluation II and sequential organ failure assessment (SOFA) scores, a New York Health Association class 3 or 4 and infection with *Acinetobacter* spp. were associated with mortality (3, 16). In another study, a higher SOFA score was found to be associated with sepsis mortality in Southeast Asia (16). The low availability of resources and inadequacy of treatment were independently associated with mortality (5).

Given the scarcity of information, especially data related to mortality and length of stay among sepsis patients admitted to the ICU in Malaysia, this study was conducted to estimate the rate of mortality and explore factors associated with ICU mortality and length of stay among ICU patients admitted for sepsis. This study will help to shed light on this issue and to limit the progress of sepsis in addition to curbing mortality, hence reducing the sepsis mortality rate and prolonged ICU stays.

## Methods

This was a prospective cohort study. All patients admitted to the ICU of Hospital Universiti Sains Malaysia (HUSM) from 1 January 2019 to 31 December 2019 who fulfilled the eligibility criteria were included in this study. The patients’ information was collected on admission and all patients were followed up until death or until discharged from ICU.

The eligibility criteria were: i) having been admitted to the general, surgical, trauma or neurosurgical intensive care units, medical high-dependency unit or coronary care unit; ii) presenting evidence of sepsis based on the Sepsis-3 criteria and iii) being 18 years old or above. Patients admitted for traumatic injuries or cardiac surgeries were excluded. The ICU was defined as any ward explicitly designed for intensive care.

The sample size requirement was calculated using the standard formula for the estimation of a population’s proportion: *n* = (Z_α_/Δ )^2^P(1−P). To approximate a 95% confidence interval (Z_α_ = 1.96), margin of error (Δ) of 7.5% and proportion of 62.8% (7), the calculated sample size was 160 patients. The sample size was inflated to account for 20% anticipated missing data and therefore, the corrected sample size was 200 patients. No sampling method was applied as all eligible patients were included in this study.

A data collection sheet was utilised to collect and record the following data: i) demographic characteristic (age and gender); ii) admission characteristics (date of admission, location and type of admission); the location of admission was categorised as the general ICU, surgical ICU (SICU), trauma ICU (TICU), neuro ICU, high-dependency unit or cardiac ICU (CCU), whereas the type of admission was based on the discipline the treatment was drawn from (medical, elective surgical admission or emergency surgical admission); iii) comorbidities; iv) clinical characteristics on admission, including mean arterial pressure, systolic blood pressure, heart rate, temperature respiratory rate and Glasgow coma scale (GCS) and SOFA scores; v) haematological and biochemical parameters on admission (total white blood cells, haemoglobin, haematocrit, platelet count, creatinine, sodium, albumin, aspartate aminotransferase, alanine aminotransferase, total bilirubin, urea, pH, arterial oxygen partial pressure (PaO_2_), fractional inspired oxygen (FiO_2_) and PF ratio; vi) treatment in ICU, including mechanical ventilation, renal replacement therapy and ionotropic/vasopressor therapy and vii) ICU status (do not resuscitate [DNR], withdrawal of therapy or withholding of therapy). All patients were followed-up until death or discharge from the ICU. ICU mortality was defined as death due to any cause and length of stay was categorised as either prolonged (more than 14 days in the ICU) or non-prolonged (equal to or less than 14 days) (17).

The data analysis was conducted in R software version 4.0.3. Descriptive statistics were generated using the *arsenal* package for all participants according to mortality (survival or death) and length of hospital stay (non-prolonged or prolonged ICU stay). All numerical variables were described as means and standard deviations (SD). For categorical variables, frequency (*n*) and column percentage (%) were calculated. Estimation of the mortality rate and proportions of patients with prolonged ICU stays were made using a binomial test to obtain point and interval estimates (with a 95% CI). Univariable (simple) and multivariable (multiple) binary logistic regression analyses were conducted to determine the factors associated with mortality and prolonged ICU stay. Variables with *P*-values of less than 0.1 in simple logistic regression were included for variable selection in the multiple logistic regression analysis. Variable selections were made using the automated likelihood ratio-based backward selection method with the *rms* package. Interaction and multicollinearity between variables were assessed before the evaluation of model fitness (via the area under the receiver operating characteristic [ROC] curve and Hosmer-Lemeshow test). The results were presented as crude odds ratios (ORs), 95% CI of crude ORs and *P*-values for simple logistic regression. For the multiple logistic regression, adjusted ORs, 95% CI of ORs and *P*-values are presented.

## Results

**Figure 1:**
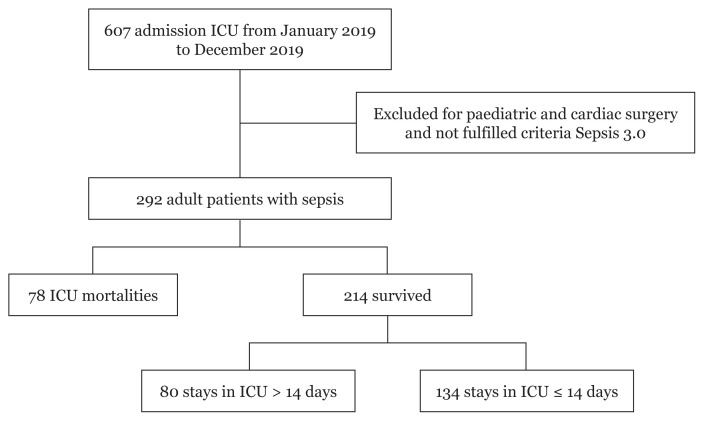
Number of ICU mortality and prolonged hospital stay among patients with sepsis who survived in HUSM 2019

A total of 292 out of 607 patients were admitted to ICU or the high-dependency unit with sepsis throughout the study period (Figure 1). The prevalence of sepsis was 48%. The baseline demographic, clinical and laboratory characteristics, and the outcomes of the 292 patients with sepsis are summarised in Table 1. The ages of the patients ranged from 18 years old to 93 years old, with a mean (SD) of 50.98 (17.75) years old. Most of the patients were male (*n* = 194, 66.4%) and admitted to the general ICU ward (*n* = 115, 39.4%), followed by the SICU (*n* = 63, 21.6%), high-dependency ward (*n* = 55, 18.8%), neuro ICU (*n* = 28, 9.6%), TICU (*n* = 26, 8.9%) and CCU (*n* = 5, 1.7%). The three most common comorbidities noted in these patients were chronic cardiovascular disease (60.6%), diabetes mellitus (51.7%) and chronic kidney disease (29.1%).

Most of the individuals were medical patients (*n* = 198, 68.0%) while for surgical patients, sepsis was more prevalent in those who underwent emergency surgery (*n* = 87, 29.9%) rather than elective surgery (*n* = 6, 2.1%). In relation to organ dysfunction, the mean SOFA score of these patients was 9.65 (SD = 4.08). At baseline, there were no apparent abnormalities in their blood pressure, temperature or respiratory rate. However, the patients were noted to have tachycardia, with a mean baseline heart rate of 102.77 (SD = 21.52) beats per min, reduced consciousness and a mean GCS value of 7.18 (SD = 4.93).

Most patients received mechanical ventilation (*n* = 260, 89.0%) and inotropic or vasopressor support (*n* = 238, 81.5%), with renal replacement therapy required in nearly one-third (*n* = 92, 31.2%). Instructions for DNR, withdrawal or withholding of therapy were in lower proportion for these patients. In terms of laboratory characteristics, the patients appeared to have anaemia (mean haemoglobin: 10.86 [SD = 2.20] g/dL), leucocytosis (mean total white blood cell (WBC): 17.17 [SD = 14.70] × 10^9^/L), impaired urea (mean urea: 13.69 [SD = 11.62] mmol/L) and creatinine (mean creatinine: 212.51 [SD = 217.89] μmol/L), hypoalbuminemia (mean albumin: 29.41 [SD = 7.25] g/dL), aspartate aminotransferase (AST) mean: 92.90 [SD = 268.37] unit/L and alanine aminotransferase (ALT) mean: 85.58 [SD = 247.84] unit/L), acidaemia (mean pH: 7.33 [SD = 0.09]) and impaired PaO_2_:FiO_2_ (mean PaO_2_:FiO_2_: 288.25 [SD = 133.97]).

### ICU Mortality

Out of 292 sepsis patients, ICU mortality was observed among 78. The mortality rate among sepsis patients admitted to the ICU in our hospital was therefore estimated to be 26.7% (95% CI: 21.7, 32.2). The time to ICU death ranged from 1 day to 63 days, with an estimated median of 14.0 (95% CI: 9.0, 18.0). Table 2 presents the results of the simple and multiple logistic regression analyses, which were performed using the automated likelihood ratio test-based backward selection method. All variables were included in the analysis, except for a few for which there were a small number of observations in each group (HIV status, immunosuppressive, haematological disorder and ICU status).

In the univariable analysis, type of admission, comorbidities (chronic kidney disease and diabetes), GCS and SOFA scores, creatinine level, albumin, ALT, bilirubin, pH, FiO_2_, PaO_2_ in mmHg to FiO_2_ expressed as a fraction (PF) ratio and treatment in ICU (the need of mechanical ventilation, renal replacement therapy and ionotropic/vasopressor) were significantly associated with ICU mortality. In the multivariable analysis, only GCS and SOFA scores were significantly associated with ICU mortality. Patients with higher GSC scores had lower odds of ICU mortality (adjusted OR = 0.90; 95% CI: 0.82, 0.98; *P* = 0.019) and patients with higher SOFA scores had higher odds of ICU mortality (adjusted OR = 1.22; 95% CI: 1.11, 1.35; *P* < 0.001).

### Prolonged ICU Stay

Out of 214 sepsis patients who survived admission to ICU, the length of ICU stays ranged from 1 to 51 days, with a mean of 14.61 (SD = 10.53) days. Eighty patients (37.4%) had prolonged ICU stays (95% CI: 30.9, 44.2). A few variables were excluded from the logistic regression analysis due to a small number of cases in each group (chronic liver disease, peptic ulcer disease, HIV, immunosuppressive or haematological disorder and ICU status).

In the univariable analysis, GCS and SOFA scores, albumin, PaO_2_, PF ratio and treatment in ICU (mechanical ventilation, renal replacement therapy and ionotropic/vasopressor) were significantly associated with a prolonged ICU stay. In the multivariable analysis, only albumin level and renal replacement therapy were significantly associated with a prolonged ICU stay. Patients with higher albumin levels had lower odds of a prolonged ICU stay (adjusted OR = 0.94; 95% CI: 0.90, 0.98; *P* = 0.006), while patients that underwent renal replacement therapy had higher odds of a prolonged ICU stay (adjusted OR = 1.25; 95% CI: 1.74, 7.12; *P* < 0.001) (Table 3).

## Discussion

Our institution practices separation of critical care units, mainly based on the type of patient and admission: we have the SICU (mostly surgical patients), TICU (for patients with trauma), neuro ICU (for neurosurgical and neuromedical patients), CCU (patients with cardiac diseases) and medical HDU (mostly medical patients). The general ICU and SICU, on the other hand, cater to all types of admission, making them the units with the greatest number of patients admitted for sepsis. General ICU and SICU admission and discharge were managed by the anaesthesiologist and intensivist in charge. The other 26 ICU beds were managed by a primary team and anaesthesiologist. Admission and discharge were controlled by the primary team. This translates to heterogeneity in the data obtained. For instance, the CCU usually admits only cardiac patients, but in our study, five patients landed up there with sepsis, contributing to the statistics.

Our study revealed that most sepsis patients were male. These findings were similar to those of a study by Mayr et al. in 2019 (18), which revealed that the elderly, males and those with multiple comorbidities were more prone to develop sepsis. Of the patients, 60.6% were admitted with cardiovascular disease, 51.7% with diabetes mellitus and 29.1% with chronic kidney disease. Even though none of the comorbidities revealed a significant association with prolonged ICU stay, the use of renal replacement therapy (RRT) was associated with mortality.

### ICU Mortality

We found that GCS and SOFA scores were significantly associated with ICU mortality. Patients with higher GCS scores on admission had lower odds of ICU mortality, whereas those with higher SOFA scores on admission had higher odds of ICU mortality.

Albumin and bilirubin levels have been reported to be associated with mortality among sepsis patients in previous studies (19–22). In 2020, a higher bilirubin-to-albumin ratio was found to be associated with mortality (23). In our study, both albumin (crude OR = 0.93, *P* < 0.001) and bilirubin (crude OR = 1.02, *P* = 0.011) were significantly associated with ICU mortality in the univariable analysis. Patients with higher albumin levels had lower odds of ICU mortality, whereas patients with higher bilirubin levels had higher odds of ICU mortality. In the multivariable analysis, however, neither variable was significantly associated with ICU mortality.

We could not prove an association between mortality and other haematological and biochemical parameters, such as platelet, sodium and AST levels. This discordant result could be related to the differences in healthcare delivery practices followed by the many physicians covering the ICUs.

### Prolonged Hospital Stays

Among the 214 sepsis patients who survived, the mean length of ICU stay in our hospital was 14.61 days, with 37.4% of patients requiring prolonged ICU stays. Albumin level and renal replacement therapy were significantly associated with a prolonged ICU stay. Patients with higher albumin levels had lower odds of a prolonged ICU stay, whereas those who required renal replacement therapy had higher odds of a prolonged ICU stay.

In this study, we did not find any association between platelet, creatinine or ALT levels and prolonged ICU stay in either the univariable or multivariable analysis. Previous studies reported that amongst the risk factors for prolonged ICU stay (defined as admission for more than 14 days) are low platelet, high creatinine, high sodium, low bilirubin and high ALT levels (19–21, 24). The pathophysiology is complex and multifactorial; it involves changes in renal haemodynamics, endothelial dysfunction, intraglomerular thrombosis and the congestion of tubules by waste and necrotic cells (25).

Other risk factors (platelet, sodium, bilirubin and ALT) were found to be insignificant, even though other studies usually found these factors to be associated with a prolonged ICU stay. Similarly, while thrombocytopaenia was found to be a significant risk factor in previous studies (26–28), we failed to demonstrate this, which could be due to the varying degrees of thrombocytopaenia not being captured during the sampling.

Ni et al. (29) found that most of their patients had hypernatremia, with a strong association with prolonged need for mechanical ventilation, weakness, and ultimately, prolonged ICU stay. However, our findings were not consistent with theirs, which could be due to the heterogeneity of the patients we treated at USM Hospital; moreover, some of the patients would only have exhibited hypernatremia after a prolonged stay. ALT has been long regarded as one of the prognostic factors for liver disease secondary to hypoxic or ischaemic hepatitis and this especially holds true for septic patients (23, 30, 31).

## Conclusion

In this study, we concluded that the prevalence of sepsis was 48%. The ICU mortality rate among sepsis patients admitted to the ICU was 26.7% and prolonged ICU stays were observed in 37.4% of sepsis patients who survived. Patients with higher GCS scores on admission had a lower probability of ICU mortality, whereas those with higher SOFA scores on admission had a higher probability of ICU mortality. Patients with higher albumin levels had a lower probability of prolonged ICU stay, whereas those who required renal replacement therapy had a higher probability of a prolonged ICU stay.

### Limitations and Strengths

Our study has some limitations. First, we had no external monitoring by an independent party that could increase the internal validity of the study. However, all the data were carefully reviewed and any extreme values were thoroughly examined to reduce errors before inputting them into the database. Second, as this study was conducted in a tertiary centre with teaching capacity, it is difficult to extrapolate these findings to the general population to determine the actual values.

This is the first study that sought to measure the ICU mortality rate and prolonged ICU stays among patients with sepsis in the critical units at Hospital USM. This prospective study gives us in-depth information about the epidemiology of sepsis in Malaysian ICUs to provide a clearer picture of septic patients in our units as well as their outcomes.

## Figures and Tables

**Table 1:** Demographic, admission characteristics, comorbidities, clinical characteristics, haematological parameters, biochemical parameters, treatment in ICU and ICU status among patients with sepsis admitted to HUSM from January to December 2019

Variables	Total (*N* = 292)	ICU mortality (*n* = 292)		Prolonged ICU stay (*n* = 214)	
	
		No (*n* = 214)	Yes (*n* = 78)	No (*n* = 134)	Yes (*n* = 80)
Demography					
Age (years old)*	50.97 (17.753)	50.05 (17.80)	53.50 (17.50)	50.22 (18.45)	49.78 (16.75)
Gender (male)	194 (66.4)	140 (65.4)	54 (69.2)	86 (64.2)	54 (67.5)
Admission characteristics					
Location					
General ICU	115 (39.4)	78 (36.4)	37 (47.4)	45 (33.6)	33 (41.2)
SICU	63 (21.6)	46 (21.5)	17 (21.8)	26 (19.4)	20 (25.0)
TICU	26 (8.9)	22 (10.3)	4 (5.1)	15 (11.2)	7 (8.8)
Neuro ICU	28 (9.6)	24 (11.2)	4 (5.1)	17 (12.7)	7 (8.8)
8S (High dependency ward)	55 (18.8)	41 (19.2)	14 (17.9)	29 (21.6)	12 (15.0)
CCU	5 (1.7)	3 (1.4)	2 (2.6)	2 (1.5)	1 (1.2)
Type of admission					
Medical	198 (68.0)	137 (64.3)	61 (78.2)	85 (63.9)	52 (65.0)
Elective surgical	6 (2.1)	5 (2.3)	1 (1.3)	4 (3.0)	1 (1.2)
Emergency surgical	87 (29.9)	71 (33.3)	16 (20.5)	44 (33.1)	27 (33.8)
Comorbidities					
Cardiovascular disease	177 (60.6)	126 (58.9)	51 (65.4)	73 (54.5)	53 (66.2)
Chronic lung disease	41 (14.0)	31 (14.5)	10 (12.8)	21 (15.7)	10 (12.5)
Chronic neurological disease	9 (3.1)	5 (2.3)	4 (5.1)	1 (0.7)	4 (5.0)
Chronic kidney disease	85 (29.1)	53 (24.8)	32 (41.0)	29 (21.6)	24 (30.0)
Chronic liver disease	4 (1.4)	1 (0.5)	3 (3.8)	1 (0.7)	0 (0.0)
Peptic ulcer disease	4 (1.4)	2 (0.9)	2 (2.6)	2 (1.5)	0 (0.0)
Diabetes	151 (51.7)	103 (48.1)	48 (61.5)	63 (47.0)	40 (50.0)
HIV	1 (0.3)	1 (0.5)	0 (0.0)	1 (0.7)	0 (0.0)
Connective tissue disease	18 (6.2)	14 (6.5)	4 (5.1)	9 (6.7)	5 (6.2)
Immunosuppressive	1 (0.3)	1 (0.5)	0 (0.0)	1 (0.7)	0 (0.0)
Homological disorder	2 (0.7)	2 (0.9)	0 (0.0)	2 (1.5)	0 (0.0)
Malignancy	25 (8.6)	21 (9.8)	4 (5.1)	13 (9.7)	8 (10.0)
Clinical characteristics					
Mean arterial pressure (mmHg)*	86.58 (15.89)	87.53 (15.82)	83.99 (15.88)	87.76 (15.37)	87.14 (16.66)
Systolic blood pressure (mmHg)*	128.79 (23.95)	130.27 (24.61)	124.73 (21.67)	130.11 (21.81)	130.53 (28.84)
Heart rate (beats/minute)*	102.77 (21.52)	102.38 (21.19)	103.84 (22.49)	102.99 (21.81)	101.36 (20.21)
Temperature (°C)*	37.12 (1.16)	37.07 (1.13)	37.27 (1.24)	37.02 (1.20)	37.15 (0.99)
Respiratory rate, (breaths/minute)*	13.87 (4.59)	13.958 (4.733)	13.645 (4.204)	14.42 (5.24)	13.19 (3.64)
GCS	7.18 (4.93)	8.10 (5.08)	4.667 (3.429)	9.09 (5.08)	6.438 (4.67)
SOFA score	9.65 (4.08)	8.75 (4.05)	12.128 (3.021)	7.87 (3.91)	10.23 (3.87)
Haematological and biochemical parameters					
Total WBC (×10^9^)*	17.17 (14.70)	16.97 (16.16)	17.71 (9.73)	16.69 (18.84)	17.435 (10.27)
Haemoglobin (g/dL)*	10.86 (2.20)	10.98 (2.13)	10.54 (2.37)	11.18 (2.07)	10.64 (2.20)
Haematocrit*	35.18 (27.36)	35.15 (28.35)	35.24 (24.59)	34.07 (6.69)	36.97 (45.68)
Platelet (×10^3^)*	198.57 (109.40)	196.50 (108.27)	204.23 (112.97)	199.58 (102.97)	191.35 (117.10)
Creatinine (μmol/L)*	212.51 (217.89)	189.34 (201.96)	276.08 (247.01)	170.65 (185.99)	220.64 (223.92)
Sodium (mEq/L)*	136.22 (10.75)	136.28 (11.68)	136.04 (7.67)	136.01 (13.03)	136.74 (9.05)
Albumin (g/dL)*	29.41 (7.25)	30.36 (7.32)	26.79 (6.39)	31.81 (6.85)	27.94 (7.48)
Aspartate aminotransferase (unit/L)*	85.58 (247.84)	59.41 (95.19)	157.39 (447.14)	53.13 (46.38)	69.94 (143.65)
Alanine aminotransferase (unit/L)*	92.90 (268.37)	60.45 (74.92)	181.91 (495.70)	55.03 (77.94)	69.54 (69.09)
Total bilirubin (μmol/L)*	22.46 (28.47)	19.21 (16.94)	31.39 (46.48)	18.59 (17.16)	20.238 (16.61)
Urea (mmol/L)*	13.69 (11.62)	13.33 (12.34)	14.71 (9.39)	12.43 (11.46)	14.83 (13.62)
Ph*	7.33 (0.09)	7.34 (0.08)	7.31 (0.12)	7.35 (0.07)	7.33 (0.09)
PaO_2_*	142.20 (65.15)	143.31 (58.94)	139.15 (80.13)	150.29 (61.73)	131.62 (52.271
FiO_2_*	0.54 (0.19)	0.51 (0.18)	0.60 (0.19)	0.50 (0.19)	0.54 (0.19)
PF ratio*	288.25 (133.97)	302.99 (130.51)	247.78 (135.84)	324.01 (134.62)	267.79 (115.79)
Treatment in ICU					
Mechanical ventilation	260 (89.0)	185 (86.4)	75 (96.2)	110 (82.1)	75 (93.8)
Renal replacement therapy	92 (31.5)	48 (22.4)	44 (56.4)	17 (12.7)	31 (38.8)
Ionotropic or vasopressor	238 (81.8)	165 (77.5)	73 (93.6)	95 (71.4)	70 (87.5)
ICU status					
Do not resuscitate	6 (2.1)	0 (0.0)	6 (7.8)	0 (0.0)	0 (0.0)
Withdrawal of therapy	2 (0.7)	0 (0.0)	2 (2.6)	0 (0.0)	0 (0.0)
With-holding of therapy	7 (2.4)	1 (0.5)	6 (7.8)	0 (0.0)	1 (1.3)

Notes: All categorical variables were presented as frequency (*n*) and column percentage (%), and all numerical variables were presented as mean and standard deviation (SD),

* mean (SD)

**Table 2:** Simple and multiple logistic regression analysis to determine factors associated with ICU mortality among patients admitted to ICU with sepsis

Variables	Crude OR	95% CI	*P-*value	Adjusted OR	95% CI	*P-*value
Demography						
Age (years old)	1.01	1.00, 1.03	0.143			
Gender (male)	1.19	0.68, 2.08	0.542			
Admission characteristics						
Location						
General ICU	ref					
SICU	0.78	0.39, 1.54	0.472			
TICU	0.38	0.12, 1.19	0.098			
Neuro ICU	0.35	0.11, 1.09	0.069			
8S (high dependency ward)	0.72	0.35, 1.48	0.372			
CCU	1.41	0.23, 8.77	0.716			
Type of admission						
Medical	ref					
Elective surgical	0.45	0.05, 3.93	0.469			
Emergency surgical	0.51	0.27, 0.94	0.032			
Comorbidities						
Cardiovascular disease	1.32	0.77, 2.26	0.315			
Chronic lung disease	0.87	0.40, 1.87	0.717			
Chronic neurological disease	2.26	0.59, 8.64	0.234			
Chronic kidney disease	2.11	1.22, 3.65	0.007			
Chronic liver disease	8.52	0.87, 83.17	0.065			
Peptic ulcer disease	2.79	0.39, 20.15	0.309			
Diabetes	1.72	1.02, 2.93	0.044			
Connective tissue disease	0.77	0.25, 2.42	0.657			
Malignancy	0.50	0.16, 1.50	0.214			
Clinical characteristics						
Mean arterial pressure, mmHg	0.99	0.97, 1.01	0.093			
Systolic blood pressure, mmHg	0.99	0.98, 1.01	0.083			
Heart rate, beats/min	1.00	0.99, 1.02	0.607			
Temperature (°C)	1.18	0.93, 1.50	0.177			
Respiratory rate (breaths/min)	0.98	0.93, 1.04	0.606			
GCS	0.83	0.77, 0.89	< 0.001	0.90	0.82, 0.98	0.019
SOFA score	1.29	1.18, 1.41	< 0.001	1.22	1.11, 1.35	< 0.001
Haematological and biochemical parameters						
Total WBC (×10^9^)	1.00	0.99, 1.02	0.706			
Haemoglobin (g/dL)	0.91	0.81, 1.03	0.132			
Haematocrit	1.00	0.99, 1.01	0.982			
Platelet (×10^3^)	1.00	0.99, 1.01	0.593			
Creatinine (μmol/L)	1.01	1.01, 1.02	0.005			
Sodium (mEq/L)	1.00	0.97, 1.02	0.865			
Albumin (g/dL)	0.93	0.90, 0.97	<0.001			
Aspartate aminotransferase (unit/L)	1.00	0.99, 1.01	0.060			
Alanine aminotransferase (unit/L)	1.00	1.00, 1.01	0.018			
Total bilirubin (μmol/L)	1.02	1.01, 1.03	0.011			
Urea (mmol/L)	1.01	0.99, 1.03	0.370			
pH	0.03	0.01, 0.45	0.011			
PaO_2_	1.00	0.99, 1.01	0.629			
FiO_2_	8.78	2.39, 32.2	0.001			
PF ratio	1.01	1.00, 1.02	0.002			
Treatment in ICU						
Mechanical ventilation	3.92	1.16, 13.25	0.028			
Renal replacement therapy	4.48	2.58, 7.76	0.000			
Ionotropic or vasopressor	4.25	1.62, 11.11	0.003			

Notes:

* Automated likelihood-ratio-test-based backward selection method applied for multiple logistic regression analysis; Area under ROC curve = 75.5% (95% CI: 69.6, 81.4); No multicollinearity and no interaction between variables; Hosmer-Lemeshow test *P*-value = 0.714,

OR = odds ratio

**Table 3:** Simple and multiple logistic regression analysis to determine factors associated with prolonged ICU stay among patients admitted to ICU with sepsis

Variables	Crude OR	95% CI	*P*-value	Adjusted OR	95% CI	*P*-value
Demography						
Age (years old)	1.00	0.98, 1.01	0.860			
Gender (male)	1.16	0.65, 2.08	0.621			
Admission characteristics						
Location						
General ICU	ref					
SICU	1.05	0.5, 2.19	0.899			
TICU	0.64	0.23, 1.74	0.377			
Neuro ICU	0.56	0.21, 1.51	0.252			
8S (high dependency ward)	0.56	0.25, 1.27	0.166			
CCU	0.68	0.06, 7.84	0.759			
Type of admission						
Medical	ref					
Elective surgical	0.41	0.04, 3.76	0.429			
Emergency surgical	1.00	0.56, 1.81	0.992			
Comorbidities						
Cardiovascular disease	1.64	0.92, 2.91	0.092			
Chronic lung disease	0.77	0.34, 1.73	0.524			
Chronic neurological disease	7.00	0.77, 63.77	0.084			
Chronic kidney disease	1.55	0.83, 2.92	0.172			
Diabetes	1.13	0.65, 1.96	0.672			
Connective tissue disease	0.93	0.3, 2.87	0.894			
Malignancy	1.03	0.41, 2.62	0.943			
Clinical characteristics						
Mean arterial pressure (mmHg)	1.00	0.98, 1.02	0.780			
Systolic blood pressure (mmHg)	1.00	0.99, 1.01	0.905			
Heart rate (beats/min)	1.00	0.98, 1.01	0.586			
Temperature (°C)	1.11	0.86, 1.44	0.426			
Respiratory rate (breaths/min)	0.94	0.87, 1.01	0.075			
GCS	0.90	0.84, 0.95	< 0.001			
SOFA score	1.17	1.08, 1.26	< 0.001			
Haematological and biochemical parameters						
Total WBC (×10^9^)	1.00	0.99, 1.02	0.746			
Haemoglobin (g/dL)	0.89	0.78, 1.01	0.074			
Haematocrit	1.00	0.99, 1.01	0.502			
Platelet (×10^3^)	1.00	0.99, 1.01	0.590			
Creatinine (μmol/L)	1.00	0.99, 1.01	0.089			
Sodium (mEq/L)	1.01	0.98, 1.03	0.660			
Albumin (g/dL)	0.93	0.89, 0.97	< 0.001	0.94	0.90, 0.98	0.006
Aspartate aminotransferase (unit/L)	1.00	0.98, 1.01	0.288			
Alanine aminotransferase (unit/L)	1.00	0.99, 1.01	0.194			
Total bilirubin (μmol/L)	1.01	0.99, 1.02	0.498			
Urea (mmol/L)	1.02	0.99, 1.04	0.180			
pH	0.08	0.01, 2.54	0.151			
PaO_2_	0.97	0.98, 0.99	0.028			
FiO_2_	3.28	0.76, 14.24	0.112			
PF ratio	0.98	0.97, 0.99	0.003			
Treatment in ICU						
Mechanical ventilation	3.27	1.2, 8.96	0.021			
Renal replacement therapy	4.35	2.21, 8.59	< 0.001	1.25	1.74, 7.12	< 0.001
Ionotropic or vasopressor	2.80	1.31, 6.00	0.008			

Notes:

* Automated likelihood-ratio-test-based backward selection method applied for multiple logistic regression analysis; Area under ROC curve = 70.3% (95% CI: 62.8%, 77.8%); No multicollinearity and no interaction between variables; Hosmer-Lemeshow test *P*-value = 0.189
